# Assessing loading rate in frequency domain by accelerometry

**DOI:** 10.1177/09544119251393825

**Published:** 2025-11-11

**Authors:** Jin Luo, Noushin Ahmadvand, Mike Crooks

**Affiliations:** 1 School of Medicine and Biosciences, University of West London, UK; 2 Right Step OÜ, Tallinn, Estonia

**Keywords:** gait, acceleration, ground reaction force, musculoskeletal load, body segments

## Abstract

The aim of the study was to develop a method to assess loading rate in the frequency domain using accelerometry, and to examine how the frequency-domain loading rate changes with body location and relates to time-domain loading rate during walking. A method was developed to calculate loading rate from acceleration signal by decomposing active motion and impact loading components in the signal into different frequency bands. The method was used to analyse an open access dataset consisting of acceleration and ground reaction force data of human walking. Acceleration data measured at pelvis, thigh, shanks, and feet during walking were used to obtain loading rate at four frequency bands: 0–3, 3–6, 6–10, and 10–15 Hz. Ground reaction forces were analysed to obtain time-domain loading rate measurements, including Average Loading Rate (ALR) and Instantaneous Loading Rate (ILR). Loading rate at all four frequency bands was attenuated significantly from foot to pelvis (*p* < 0.001). However, the pattern of attenuation was different at low frequency bands (below 10 Hz) compared to high frequency bands (above 10 Hz). Loading rate measured at body segments in the frequency domain was significantly correlated with ALR and ILR (*R*^2^ from 0.44 to 0.56). However, the strength of correlation was higher in low frequency bands (below 10 Hz) than high frequency bands (above 10 Hz). The study suggests that assessing loading rate in the frequency domain can provide additional insights into the load experienced by specific body segments in human locomotion.

## Graphical abstract

**Graphical abstract:**
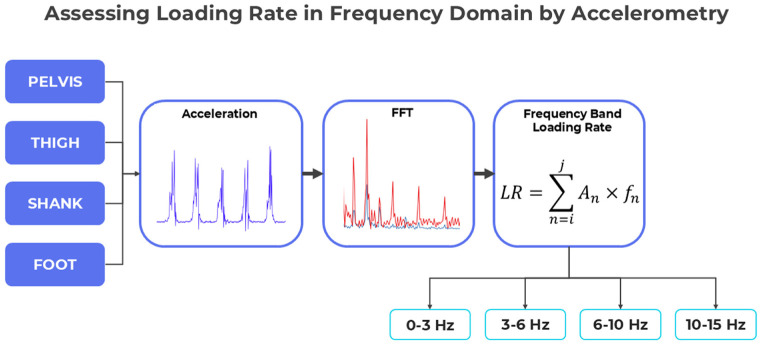

## Introduction

Appropriate levels of mechanical load are crucial for musculoskeletal health. Lack of loading may lead to diseases such as osteoporosis and sarcopenia, while excessive mechanical loading is associated with injuries and osteoarthritis. It is thus important to assess and monitor musculoskeletal load in real time and in natural environments. To this end, innovative technologies of wearable sensors have been developed by previous research to assess musculoskeletal load.^1^

Biomechanical parameters are often used as biomarkers to quantify musculoskeletal load. Among them, loading rate is an important biomarker that measures how fast a load changes with time. Previous research found that loading rate was directly linked to biological responses of musculoskeletal tissues. Animal experiments showed that bone formation rate was proportional to strain rate of dynamic loading,^2^ while observational studies on human indicated that loading rate was associated with tibia stress fracture,^3^ knee osteoarthritis,^4^ and recovery progress from hip fracture.^5^

To date, loading rate is mainly assessed in the time domain based on the measurement of ground reaction force.^6^ However, time-domain analysis may not provide comprehensive information as the biological effect of loading rate varies at different frequency bands.^7,8^ For example, the osteogenic response of cortical bone to loading rate was non-linear over a frequency range between 1 and 30 Hz, with the highest response at 5–10 Hz.^7,9^ On the other hand, loading rate above 10 Hz was found to be associated with running-related injury risk.^10^ Although loading frequency has been used as a key parameter for quantifying musculoskeletal load,^11,12^ the separation of its effect into specific frequency bands might be able to provide more clinically relevant information.^10,13^

It was found that ground reaction force (GRF) of human locomotion consists of two main frequency components, a low frequency one below 10 Hz and a high frequency one greater than 10 Hz. The low frequency component was associated with active force during stance phase, while the high frequency component was associated with impact loading during stance phase.^13^ As the two components occur simultaneously, their effects on loading could not be assessed separately using time domain analysis. However, the two components propagate differently through the body and therefore may induce different effects on body segments.^14^ The separation of these components is therefore beneficial to understand how active force and impact loading in human locomotion could have their specific effects on performance and risk of injury.^15^ The analysis of loading rate in the frequency domain may provide a way to disentangle these components and shed more insight on musculoskeletal loading. Further analysis can be conducted using wavelet transformation to examine how the frequency components change at specific time points during gait cycles.^16^

The human body is a complex mechanical system. Although external load is mainly applied through the contact between feet and ground during human locomotion, it has to be transmitted through the body, which could cause amplification or attenuation of the load signal through passive mechanisms via bone and soft tissues^17^ and active mechanisms due to muscle contraction and limb motion.^18^ The amplification or attenuation of GRF signal may be different for active force component and impact loading component as the aforementioned mechanisms work most effectively at different frequency ranges.^17^ Because of this, loading rate assessed from ground reaction force is not necessarily an accurate predictor of loading rate at other parts of the body.^19^ Therefore, it would be beneficial to assess loading rate at specific body segments using accelerometry.

The first aim of the current study was to develop a method to assess loading rate at frequency bands corresponding to active motion and impact loading components using accelerometry during walking. Secondly, we aimed to examine how the frequency-domain loading rate measurements changed with body locations during walking. Our third aim was to correlate the frequency-domain loading rate measurements with the time-domain loading rate measurements. We hypothesised that loading rate changes with body location during human locomotion, and the pattern of change would be different for different frequency bands.

## Methods

### Analysis of loading rate in the frequency domain by accelerometry

A time-domain acceleration signal A(t) satisfying Dirichlet conditions can be expressed by discrete Fourier transformation (DFT) as the sum of a finite number of sine and cosine components:

(1) $$A\left(t\right)=a_{0}+\sum_{n=1}^{N/2}(a_{n}\cos\left(n\omega t\right)+b_{n}\sin(n\omega t))\;$$

where N is the total number of points for A(t) , n represents the nth harmonic; $a_{n}$ and $b_{n}$ are harmonic coefficients, ω represents the fundamental frequency of the signal, and t is the time. The magnitude of each harmonic for A(t) is defined as

(2) $$A_{n}=\sqrt{a_{n}^{2}+b_{n}^{2}}$$

The rate of change of the acceleration signal A(t) can be obtained by differentiation of equation (1) with respect to t ,

(3) $$\overset{\cdot }{A}(t)=\sum_{n=1}^{N/2}(-n\omega a_{n}\sin(n\omega t)+n\omega b_{n}\cos(n\omega t))\;$$

where $\overset{\cdot }{A}(t)$ represents the rate of change. The magnitude of each harmonic for $\overset{\cdot }{A}(t)$ can be expressed as

(4) $$DA_{n}=n\omega \sqrt{a_{n}^{2}+b_{n}^{2}}=n\omega A_{n}$$

where $DA_{n}$ is the magnitude of nth harmonic of $\overset{\cdot }{A}(t)$ , which equals the magnitude $\left(A_{n}\right)$ of the harmonic of A(t) multiplied by its frequency (nω) . This provides a way to assess loading rate in the frequency domain without the need to conduct numerical differentiation of the original acceleration signal. The following equation can then be used to examine loading rate over a particular frequency band

(5) $$\mathrm{LR}=\sum_{n=i}^{j}A_{n}\times f_{n}$$

where LR (*BW*/*s*) represents the loading rate over a frequency band from *i*^th^ to *j*^th^ harmonics, $A_{n}$ is the acceleration (*g*) at the nth harmonic, and $f_{n}$ is the nth harmonic frequency (*Hz*).

### Dataset

**Figure 1.**
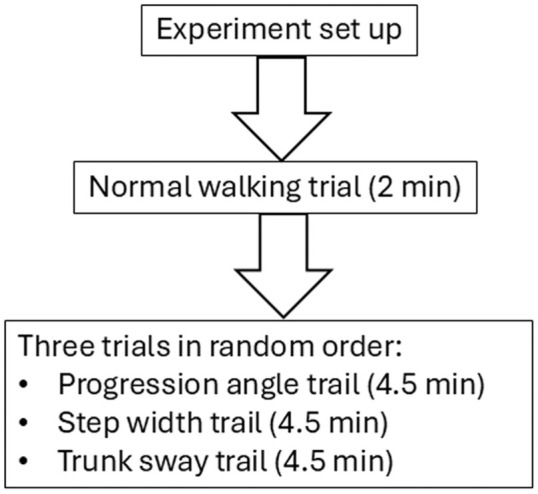
Experiment procedure.

An open access dataset^20^ was used for this study. The dataset was from an experiment of 17 healthy participants (all male; age: 23.2 ± 1.1; height: 1.76 ± 0.06 m; mass: 67.3 ± 8.3 kg; BMI: 21.5 ± 2.1 kg/m^2^). The procedure of the experiment is shown in Figure 1. Firstly, eight IMU sensors (SageMotion, Kalispell, MT, USA) to measure acceleration at sampling frequency of 100 Hz were attached to trunk at midway between sternum jugular notch and sternum xiphisternal joint, to pelvis at midway between left and right anterior superior iliac spine, to both thighs at midway between anterior superior iliac spine and femur medial epicondyle, to both shanks at midway between femur medial epicondyle and tibia apex of medial malleolus, and to both feet at second metatarsal. The *z*-axes of the IMU sensors were aligned with the segment surface normal, *y*-axes were pointing upwards, and *x*-axes were perpendicular to the *y*- and *z*-axes by the right-hand rule. An instrumented treadmill with two split belts (Bertec Corp., Worthington, OH, USA) was set up to measure ground reaction force at sampling frequency of 1000 Hz. Subjects then conducted a normal walking trial on the treadmill with self-selected speeds (1.16 ± 0.04 m/s) to determine baseline progression angle and step width for three subsequent trails that were conducted in random order (Figure 1). In progression angle trial, subjects were asked to walk with the combination of three speeds (self-selected, self-selected minus 0.2 m/s, and self-selected plus 0.2 m/s) and three foot progression angles (baseline, baseline −15°, and baseline +15°). In step width trial, participants were asked to walk with the combination of the same three speeds and three step width (baseline, baseline −0.054 m, and baseline +0.070 m). In trunk sway trial, participants were asked to walk with the combination of the same speeds and three trunk sway angles (4°, 8°, 12°). Each combination lasted for 30 s, and was conducted in random order in each trial that lasted for 4.5 min (30 s × 9 combinations). As participants walked for various number of steps ranging from 600 to 900, the data for the first 600 steps were analysed for each participant. In this study we used the acceleration data measured on the pelvis, right thigh, right shank, and right foot, and the ground reaction force measured on the right leg in each step. Ground reaction force data were low-pass filtered at 15 Hz after collection.

### Data analysis and reduction

Ground reaction force and acceleration data in stance phase were analysed. Acceleration data at *x*, *y*, and *z* axis of each stance phase was low-pass filtered at 15 Hz using a fourth order Butterworth filter to match with the filtering of ground reaction force data. To improve the frequency resolution of DFT, acceleration data from 10 consecutive stance phases were concatenated at each axis. The length of the concatenated signal ranged from 5.04 to 7.97 seconds. As the result of concatenation, 600 steps of data were divided into 60 step groups.

**Figure 2.**
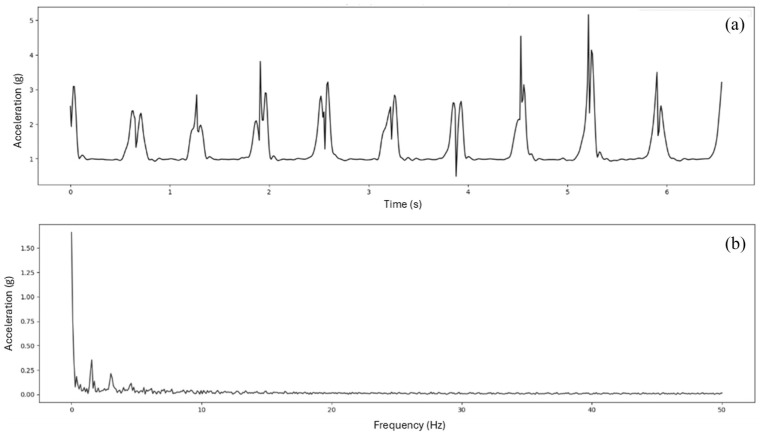
Concatenated acceleration signal of 10 stance phases measured at foot (a) and its FFT spectrum (b).

Resultant acceleration (*g*) was calculated at each of the four sensor locations (i.e. pelvis, thigh, shank, and foot) for the 10 concatenated stance phases. Fourier transformation was then performed on the concatenated acceleration data (Figure 2). Frequency-domain loading rate was then calculated using equation (5) over four frequency bands: 0–3 Hz as LR_B1, 3–6 Hz as LR_B2, 6–10 Hz as LR_B3, and 10–15 Hz as LR_B4. These frequency bands were chosen based on previous findings that acceleration during walking is composed of a low frequency component (below 10 Hz) resulting from active motion, and a high frequency component (between 10 and 20 Hz) resulting from impacts.^14^ LR_B4 (10–15 Hz) reflects the loading rate related to the impact component, while the active component was further divided into LR_B1 (0–3 Hz), LI_B2 (3–6 Hz), and LR_B3 (6–10 Hz) as the main frequency content of kinematic data for walking was found to be below 3 Hz.^21^

Frequency-domain loading rate was also calculated using a conventional time-domain approach.^22^ Acceleration data in each stance phase was band-pass filtered at four frequency bands: 0–3, 3–6, 6–10, and 10–15 Hz. Numerical differentiation was then applied to the filtered acceleration data. The differentiation values across the 10 stance phases were averaged to obtain loading rate at the four frequency bands: LR_B1_T (0–3 Hz), LR_B2_T (3–6 Hz), LR_B3_T (6–10 Hz), and LR_B4_T (10–15 Hz).

Resultant ground reaction force (rGRF) was normalised to body weight (BW). Average Loading Rate (ALR) was calculated as the slope of the line connecting the 20% and 80% points of peak rGRF from heel strike to impact peak. Instantaneous Loading Rate (ILR) was calculated as the highest first derivative of rGRF in the region between 20% and 80% of peak rGRF from heel strike to impact peak.^6^ Time-domain loading rate measurements (i.e. ALR and ILR) were calculated for individual stance phase, and were then averaged across the 10 concatenated stance phases to obtain ALR and ILR for a step group.

Frequency-domain loading rate was also calculated on the concatenated rGRF data by using equation (5), which resulted in GRF based loading rates at four frequency bands: LR_B1_GRF (0–3 Hz), LR_B2_GRF (3–6 Hz), LR_B3_GRF (6–10 Hz), and LR_B4_GRF (10–15 Hz).

### Statistical analysis

As the experimental data involve repeated measurements of GRF and acceleration at four sensor locations and longitudinal measurements of 60 step groups for each participant, General Estimating Equations (GEE) in SPSS (v28.0, Microsoft, USA) was employed to examine how sensor location affects loading rate at each frequency band. Linear model was selected for the analysis. The dependent variable was set as LR_B1, LR_B2, LR_B3, or LR_B4, while the within-subject variables included sensor location set as the factor and step group set as the covariate. If a significant effect of location was found, pairwise comparisons with Bonferroni correction were used to examine the difference between specific sensor locations.

As each participant was measured at 60 step groups on GRF and acceleration, fixed effect regression for repeated measures was employed to examine the correlation between time-domain loading rate parameter (i.e. ALR or ILR) as the dependent variable and frequency-domain loading rate parameters based on acceleration (i.e. LR_B1, LR_B2, LR_B3, and LR_B4) or based on ground reaction force (i.e. LR_B1_GRF, LR_B2_GRF, LR_B3_GRF, and LR_B4_GRF) as the independent variables. Hierarchical regression analysis was employed, with regression model 1 includes participant as the independent variable, while regression model 2 includes both participant and frequency-domain loading rate parameters as the independent variables. The change in *R*-squared from model 1 to model 2 reflects the increment in *R*-squared for frequency-domain loading rate parameters after accounting for all between-subject variation as well as other unmeasured variables that may be associated with dependent variable. A squared multiple partial *R* was calculated using the following equation to determine the percentage of the remaining unexplained variations in the dependent variable that is accounted for by frequency-domain loading rate parameters after residualising for the between-subject variation.^23,24^

(6) $$\mathrm{Multiple}\;R_{\mathrm{partial}}^{2}=\frac{R_{\mathrm{model}2}^{2}-R_{\mathrm{model}1}^{2}}{1-R_{\mathrm{model}1}^{2}}$$

Repeated measures correlation analysis were conducted to examine the relationship between loading rate calculated using equation (5) (i.e. LR_B1, LR_B2, LR_B3, and LR_B4) and that using the conventional time-domain approach (i.e. LR_B1_T, LR_B2_T, LR_B3_T, and LR_B4_T).

Significance was accepted at *p* < 0.05. Statistical analysis was performed using SPSS (v28.0, Microsoft, USA) and Python (version 3.11.8).

## Results

The mean average loading rate (ALR) calculated from the 600 steps of 17 participants was 9.14 BW/s (SD: 3.62), while the mean instantaneous loading rate (ILR) was 16.17 BW/s (SD: 4.85).

Loading rate at each frequency band changed significantly with sensor locations (*p* < 0.001). LR_B1 and LR_B2 were the highest at the foot (Table 1). Both decreased significantly at the shank and thigh, and then further decreased significantly at the pelvis (*p* < 0.05). LR_B2 was not significantly different between the shank and the thigh (*p* > 0.05). Similarly, LR_B3 and LR_B4 were the lowest at the pelvis. However, LR_B3 and LR_B4 at the thigh were not significantly decreased from the foot (*p* > 0.05). LR_B3 and LR_B4 at the thigh were also significantly higher than shank (*p* < 0.05). These results indicate that the transmission of loading rate from foot to pelvis has different patterns between low frequency bands (i.e. 0–3 and 3–6 Hz) and high frequency bands (i.e. 6–10 and 10–15 Hz).

**Table 1.** Frequency-domain loading rate parameters (BW/s) measured at different sensor locations (mean ± SE).

Loading rate	Foot	Shank	Thigh	Pelvis
LR_B1	3.19 ± 0.06	2.66 ± 0.04^a^	2.45 ± 0.05^a,b^	2.09 ± 0.03^a,b,c^
LR_B2	7.08 ± 0.25	3.80 ± 0.08^a^	3.39 ± 0.18^a^	2.79 ± 0.08^a,b,c^
LR_B3	9.89 ± 0.26	5.55 ± 0.15^a^	9.02 ± 0.41^b^	5.46 ± 0.32^a,c^
LR_B4	14.83 ± 0.54	8.58 ± 0.33^a^	17.64 ± 1.09^b^	4.59 ± 0.19^a,b,c^

LR_B1: loading rate at frequency band of 0–3 Hz; LR_B2: loading rate at frequency band of 3–6 Hz; LR_B3: loading rate at frequency band of 6–10 Hz; LR_B4: loading rate at frequency band of 10–15 Hz; BW: body weight.

a Significantly different compared with foot (p < 0.05).

b Significantly different compared with shank (p < 0.05).

c Significantly different compared with thigh (p < 0.05).

Frequency-domain loading rate parameters calculated using equation (5) was significantly correlated with that calculated using the conventional time-domain approach (*p* < 0.001; Table 2).

**Table 2.** Correlation coefficients (r) between frequency-domain loading rate parameters calculated using equation (5) and that using the conventional time-domain approach.

Sensor location	*r* (LR_B1 vs LR_B1_T)	*r* (LR_B2 vs LR_B2_T)	*r* (LR_B3 vs LR_B3_T)	*r* (LR_B4 vs LR_B4_T)
Foot	0.478***	0.405***	0.329***	0.419***
Shank	0.715***	0.455***	0.605***	0.584***
Thigh	0.698***	0.561***	0.529***	0.764***
Pelvis	0.853***	0.613***	0.751***	0.755***

LR_B1, LR_B2, LR_B3, LR_B4: loading rate at four frequency bands calculated using equation (5); LR_B1_T, LR_B2_T, LR_B3_T, LR_B4_T: loading rate at four frequency bands calculated using the conventional time-domain approach.

*** p < 0.001.

Frequency-domain loading rate parameters were significantly correlated with time-domain loading rate parameters obtained from GRF (i.e. ALR and ILR), with the squared multiple partial R ranging from 0.436 to 0.558 (Tables 3 and 4). Among the four frequence-domain loading rate parameters, LR_B1 and LR_B4 consistently showed positive and significant correlations with ALR and ILR at the four sensor locations, except at the foot where LR_B2 had significant and positive correlation (*p* < 0.01). In general, the standardised beta was the highest for LR_B1, followed by LR_B4, indicating that loading rate at these two frequency bands (i.e. 0–3 and 10–15 Hz) had the strongest correlation with time-domain loading rate parameters ALR and ILR.

**Table 3.** Regression coefficients (standardised beta) and R-squared values for regression between ALR and frequency-domain loading rate parameters at different sensor locations.

Sensor location	LR_B1 (BW/s)	LR_B2 (BW/s)	LR_B3 (BW/s)	LR_B4 (BW/s)	$R_{\mathrm{model}2}^{2}-R_{\mathrm{model}1}^{2}$	$\mathrm{Multiple}\;R_{\mathrm{partial}}^{2}$
Foot	−0.060*	0.667**	−0.101**	0.151**	0.226**	0.436
Shank	0.437**	0.084*	0.008	0.089**	0.228**	0.440
Thigh	0.531**	−0.026	−0.187**	0.222**	0.240**	0.463
Pelvis	0.397**	−0.080*	−0.002	0.318**	0.248**	0.479

ALR: average loading rate; LR_B1: loading rate at frequency band of 0–3 Hz; LR_B2: loading rate at frequency band of 3–6 Hz; LR_B3: loading rate at frequency band of 6–10 Hz; LR_B4: loading rate at frequency band of 10–15 Hz; BW: body weight.

* *p* < 0.05; **p < 0.01.

**Table 4.** Regression coefficients (standardised beta) and R-squared values for regression between ILR and frequency-domain loading rate parameters at different sensor locations.

Sensor location	LR_B1 (BW/s)	LR_B2 (BW/s)	LR_B3 (BW/s)	LR_B4 (BW/s)	$R_{\mathrm{model}2}^{2}-R_{\mathrm{model}1}^{2}$	$\mathrm{Multiple}\;R_{\mathrm{partial}}^{2}$
Foot	−0.128**	0.770**	−0.022	0.101**	0.290**	0.506
Shank	0.381**	0.142**	0.064*	0.153**	0.267**	0.466
Thigh	0.495**	−0.005	−0.143**	0.398**	0.300**	0.524
Pelvis	0.427**	−0.081**	0.153**	0.338**	0.320**	0.558

ILR: instantaneous loading rate; LR_B1: loading rate at frequency band of 0–3 Hz; LR_B2: loading rate at frequency band of 3–6 Hz; LR_B3: loading rate at frequency band of 6–10 Hz; LR_B4: loading rate at frequency band of 10–15 Hz; BW: body weight.

* *p* < 0.05; **p < 0.01.

GRF based loading rate (i.e. LR_B1_GRF, LR_B2_GRF, LR_B3_GRF, and LR_B4_GRF) were significantly correlated with ALR and ILR (Table 5). The pattern of correlation was similar as the acceleration based loading rate measured at foot (Tables 3 and 4), as both LR_B2 and LR_B2_GRF had strong correlation with ALR and ILR. On the other hand, this pattern was different from that measured at shank, thigh, and pelvis where LR_B1, instead of LR_B2, had the strongest correlation with ALR and ILR.

**Table 5.** Regression coefficients (standardised beta) and R-squared values for regression between ALR and ILR and frequency-domain loading rate parameters calculated from GRF.

Time-domain loading rate	LR_B1_GRF (BW/s)	LR_B2_GRF (BW/s)	LR_B3_GRF (BW/s)	LR_B4_GRF (BW/s)	$R_{\mathrm{model}2}^{2}-R_{\mathrm{model}1}^{2}$	$\mathrm{Multiple}\;R_{\mathrm{partial}}^{2}$
ALR	−0.164**	0.410**	−0.008	0.309**	0.323**	0.624
ILR	−0.181**	0.247**	0.067**	0.554**	0.471**	0.822

ALR: average loading rate; ILR: instantaneous loading rate; LR_B1_GRF: loading rate at frequency band of 0–3 Hz; LR_B2_GRF: loading rate at frequency band of 3–6 Hz; LR_B3_GRF: loading rate at frequency band of 6–10 Hz; LR_B4_GRF: loading rate at frequency band of 10–15 Hz; BW: body weight.

* *p* < 0.05; **p < 0.01.

## Discussion

The current study developed a method to assess loading rate in the frequency domain at different body locations by accelerometry. We found that loading rate was attenuated when transmitted from foot to pelvis, but the pattern of attenuation was different at different frequency bands. We also found that loading rate measurements in the frequency domain by accelerometry were significantly correlated with time-domain loading rate measurements by ground reaction force during walking, but the strength of correlation also depends on frequency bands. Our findings highlight the importance of assessing loading rate in the frequency domain and at different body locations using accelerometry.

Our method of assessing loading rate has several strengths. Firstly, it enables the decomposition of the effects of active motion and impact loading components on loading rate, which could not be revealed by the time domain analysis. This facilitates more clinically relevant assessments of human performance and risk of injury in various activities of human locomotion. The proposed method appears to be valid as the loading rates calculated using our method correlate significantly with those calculated from a conventional time domain approach using bandpass filter and digital differentiation. However, our method may be more convenient to implement due to the simplicity in data processing, as it does not require the detection of acceleration peaks during gait cycle or the use of bandpass filter and digital differentiation to calculate loading rate, making it an attractive method to be used in natural environment for short term or long term monitoring of musculoskeletal load.

Previous studies have used accelerometers to measure loading rate in the time domain at different body locations. Most of these studies used peak acceleration to quantify loading rate. Their findings showed that peak acceleration measured at the foot and the distal tibia had moderate to excellent positive correlations with ALR and ILR, with correlation coefficients ranging from 0.33 to 0.94.^25–27^ A recent study calculated loading rate from acceleration using numerical differentiation at different body locations such as shoulder, wrist, and hip.^22^ Moderate correlation was found between loading rate measured from accelerometers and from a force plate, with R-squared values ranging from 0.21 to 0.50 when between-subject variation was not considered. Comparable to the previous studies, our study also found that loading rate measured by accelerometers had significant correlation with ALR and ILR, with the squared multiple partial R ranging from 0.436 to 0.558. However, a major difference of our study is the assessment of loading rate in the frequency domain, which may provide more insights of musculoskeletal load at different body locations in locomotion.

The analysis of ground reaction force in the frequency domain has revealed that more than 95% of GRF signal amplitude in walking is contained within the frequency range below 20 Hz,^28^ which consists of two main components, a low frequency one below 10 Hz associated with active motion and a high frequency one greater than 10 Hz associated with impact loading during stance phase.^13^ Time-domain loading rate measurements such as ALR and IVR reflect the combined effect of both components. This can explain our results that ALR and ILR had significant correlations with loading rate measured both below 10 Hz and above 10 Hz.

Similar to ground reaction force, acceleration signal measured on human body during walking was also composed of two main frequency components, that is, a low frequency one below 10 Hz associated with the active motion and a high frequency one above 10 Hz associated with impact.^14,29,30^ The two components in acceleration are results of the transmission of the two frequency components in ground reaction force through human body. However, previous research found that the transmission characteristics were different for the two components, with the magnitude of the high frequency component of acceleration being attenuated more than the low frequency component during running.^14,18^ Our results also showed that the attenuation of loading rate from foot to pelvis had different patterns at low frequency bands (i.e. LR_B1 and LR_B2) compared to high frequency bands (i.e. LR_B3 and LR_B4), but the pattern was different from previous research. For example, there was no significant attenuation of loading rate at thigh at high frequency bands, while significant attenuation was found in low frequency bands. This may be due to the reason that loading rate is different from loading magnitude that was assessed in previous research.^14,18^

The current study also found that AVR and ILR were significantly correlated with loading rate at the low frequency band (i.e. LR_B1) measured at body segments, but had weaker correlation with loading rate at high frequency band (i.e. LR_B4). This can be explained by the fact that ground reaction force reflects the vector sum of the accelerations of all the body’s segments. The low frequency component of ground reaction force is associated with active motion of the body segments, while the high frequency component is associated with impact loading.^31^ Due to the different transmission characteristics of these frequency components through the body, ground reaction force measurement may not be able to provide an accurate estimation of loading rate at different frequency bands for body segments. This argument is supported by our results that the GRF based loading rates (i.e. LR_B1_GRF, LR_B2_GRF, LR_B3_GRF, and LR_B4_GRF) correlated with ALR and ILR in a similar pattern as acceleration based loading rate (i.e. LR_B1, LR_B2, LR_B3, and LR_B4) measured at foot, but in a different pattern from shank, thigh, and pelvis. These results demonstrated that the frequency content of mechanical loads changes during its transmission from the point of contact at foot-ground interface through the body. Our findings suggest that loading rate at specific body segments depends on the different transmission characteristics of the low frequency and high frequency components through the body, which could be revealed by assessing loading rate in the frequency domain using accelerometry to provide additional insight into the understanding of musculoskeletal load in human locomotion.

There are some limitations in this study. Firstly, the ground reaction force data in the open access dataset had been low-pass filtered at 15 Hz. This might have caused some loss of ground reaction force signal, as 95% of GRF amplitude during walking was represented within the frequency range up to around 20 Hz.^28^ We only analysed the acceleration data during the stance phase, as we were interested in how loading rate changed when it transmitted from the point of ground contact through the body. However, this does not represent the situation in real life where body segments are loaded during both stance phase and swing phase. Loading rate should be measured and analysed continuously with both stance phases and swing phases in natural environment, while ground reaction force can only measure loads in stance phase.

The method developed in the current study could have a wide range of applications in areas such as human space exploration, military, healthcare, and sports where assessing loading rate is important for preventing injury, maintaining skeletal health, monitoring disease progression, and rehabilitating patients after surgery. Our future research will examine how musculoskeletal health is associated with loading rate in the frequency domain assessed in natural environment.

## Conclusions

The current study has developed a method to assess loading rate in the frequency domain using accelerometry. Loading rate during walking was assessed at four frequency bands (0–3, 3–6, 6–10, and 10–15 Hz) and at four body locations (foot, shank, thigh, and pelvis). Loading rate changes significantly when transmitted from foot to pelvis, but the pattern of change was different between low frequency bands (below 10 Hz) and high frequency bands (above 10 Hz). Frequency-domain loading rate measurements at different body locations were significantly correlated with time-domain loading rate measurements from ground reaction force. However, the strength of correlation was higher in the low frequency bands (below 10 Hz) than the high frequency bands (above 10 Hz). Our findings suggest that assessing loading rate in the frequency domain can provide additional insights into the load experienced by specific body segments in human locomotion.
